# The Nervous System as a Regulator of Cancer Hallmarks: Insights into Therapeutic Implications

**DOI:** 10.3390/cancers14184372

**Published:** 2022-09-08

**Authors:** Karla V. Torres-Juárez, Felisbina Luisa Queiroga, Laura P. Romero-Romero

**Affiliations:** 1 Department of Pathology, Faculty of Veterinary Medicine and Zootechnics, National Autonomous University of Mexico (UNAM), CDMX 04510, Mexico; 2 Department of Veterinary Sciences, University of Trás-os-Montes and Alto Douro, 5000-801 Vila Real, Portugal; 3 Animal and Veterinary Research Centre (CECAV), University of Trás-os-Montes and Alto Douro, 5000-801 Vila Real, Portugal; 4 Center for the Study of Animal Sciences, CECA-ICETA, University of Porto, 4099-002 Porto, Portugal; 5 Associate Laboratory for Animal and Veterinary Science (AL4AnimalS), 5000-801 Vila Real, Portugal

**Keywords:** nervous system, cancer hallmarks, tumor innervation, neurotumoral communication, immunity neuromodulation

## Abstract

**Simple Summary:**

The nervous system communicates with the whole organism, regulating several physiological pathways. The modification of nerve activity could deregulate the state of cellular and tissue homeostasis which could drive cancer development. This paper provides the current state of knowledge, in an evidence-oriented manner, that the nervous system is able to participate in the carcinogenesis process by inducing biochemical, physiological, and cellular modifications involved in the hallmarks of cancer.

**Abstract:**

The involvement of the nervous system in the development of cancer is controversial. Several authors have shown opinions and conflicting evidence that support the early effect of the nervous system on the carcinogenic process. For about a century, research has not been enough, questions remain open, ideas are not discarded, and although more research is still needed to answer all the questions, there is now enough evidence to support the theories and give hope of finding one more possible form of treatment. It is clear that malignant neoplasms have endogenous characteristics that allow them to establish and progress. Some of these characteristics known as hallmarks of cancer, are damage mechanisms in the pathology but necessary during other physiological processes which show some nerve dependence. The nervous system communicates with the whole organism, regulating physiological processes necessary to respond to external stimuli and for the maintenance of homeostasis. The modification of nerve activity could generate an overload and deregulate the state of cellular and tissue homeostasis; this could drive cancer development. In this review, we will address the issue in an evidence-oriented manner that supports that the nervous system is able to participate in the initial and progressive process of carcinogenesis by inducing biochemical, physiological, and cellular modifications involved in the hallmarks of cancer.

## 1. A Bit of History

The neurobiology of cancer is not a new concept. For a long time, it was claimed that tumors were devoid of innervation, but in the nineteenth century, Young [1], through histological preparations of impregnation with methylene blue, managed to observe nerve fibers in the parenchyma of different tumor types, such as mammary carcinomas and small round cell sarcomas. At that time, the accepted belief was that the nerves found in neoplastic tissues were there due to their entrapment from their pre-existence in healthy tissue. The transformation of healthy tissue and the accelerated cellular proliferation of the malignant cells would cause the invasion of adjacent tissues and thus confine the nerves within the neoplastic tissue. This being the case, when nerves are deprived of their function and invaded by neoplastic cells, they would eventually atrophy and disappear. In 1933, Ryrie [2] explained different theories about the anatomical and physiological relationship of nerve fibers in a neoplasm. His work highlights the thought of the existence of nerve fibers in tumors, the possibility that these are newly formed, perineural invasion (PI), fibrosis after damage as a result of PI and neural regeneration similar to peripheral damage within the neoplastic tissue. However, he concluded that there is no reason to assume any trophic influence on malignant cells [2]. In 1949, Shapiro published the results of his research regarding the relationship between nerves and tumor cells, and their possible physiological role in mesotheliomas and carcinomas in the anterior chamber of the eye, transplanted in mice and rabbits, respectively. In his conclusion, he supports the suggestion that the variation in the growth rate of tumors may be due to vasomotor changes caused by the alteration in the innervation present in each tumor [3].

On the other hand, during the 1950s, some studies were conducted to show the relationship between psychosomatic problems and the development of the disease. The organism is considered as a whole, so psychosocial problems may be more associated with the cause than with the effect of the disease, which is why it is suggested that the personality structure could play a role in the pathogenesis of cancer in individuals predisposed [4]. Psychological therapy, until then, was little used as a palliative practice in cancer patients and when exploring the benefit of intensive deep psychotherapy, a significant reduction in the size of the tumors presented by the patients was reported, when recognizing and working on traumatic events of their past [5]. On the contrary, it has been seen that patients suffering from cancer and manifesting problems of depression, anxiety, chronic stress, or social isolation, have poor prognoses, which include an accelerated progression of the disease, higher rates of recurrences, and low response to treatments, compared to those patients who do not have these disorders [6,7]. In other studies, when examining the association of social stress with relapse and mortality rates in some hormone-sensitive or lifestyle-related tumors, these have been found to be increased mainly in patients with breast [6], colorectal, pancreatic [8], and gastric tumors [7], a fact that is currently being corroborated in experimental works in vivo [9,10].

Until now, the role of the emotional factors in the carcinogenesis process is not entirely clear, however, the balance seems to tilt towards active participation in the cancer initiation and progression [11,12]. 

Some authors support that cancer is a disease of cellular differentiation, communication and tissue organization [13,14], and the environmental factor is described as one of the most important elements in the progression stage. The emotional state, in many cases, depends on the environment and has the ability to generate important biological modifications which can cause the prolonged release of molecules that could stimulate the formation of a tumor due to the signals it transmits and the processes it activates [10]. In addition, modifications or interruptions in the interactions between the nervous system and tissues are likely to transform the microenvironment and create the appropriate conditions for tumor development [9]. 

Over the years, cancer research has taken a reductionist approach, neglecting the complex functioning of a complete organism with all its component parts. Until now, the cancer study model has excluded important mechanisms, such as cellular communication through gap junctions, stem cells, mechanical damage [13,15], dysbiosis [16,17], ion channels [18,19,20], and the neurobiology of the oncological process. 

## 2. Emotional State, Personality and Cancer 

Several studies support that people who present different stress-generating situations, such as a hostile family environment, a compromised economic situation or conditions of neglect or isolation, present accelerated progression of the disease with an ineffective response to treatment [6,21]. On the other hand, in different studies in which patients, in addition to pharmacological treatment, receive group and/or individual emotional therapy, coupled with physical exercise, or another type of social support and in some cases nutritional control, it was possible to observe an evident improvement, obtaining favorable results with a better response to treatments, prolonging disease-free survival, and notably improving quality of life [22,23]. Similarly, it has been proposed that character and personality, which define how we face adversity, could predispose to the development of breast cancer in women [24]. Studies in animals have produced results that support the fact that type of behavior at an early age causes differences in the production of endogenous glucocorticoids associated with the variation in the natural function of the Hypothalamic Pituitary Adrenal (HPA) axis, causing oscillation in the function of the immune system and generating individuals potentially susceptible to specific processes of disease at the end of life [25]. Therefore, if personality and character can predispose to the appearance of diseases due to the variations it causes in the functioning of the immune system, generally causing a suppressive effect, this could be the basis of the individual differences in the disease stages. 

## 3. Participation of the Nervous System in Physiological Proliferative Processes

In support of the concept that the nervous system could play a central role in the pathophysiology of cancer, there is evidence that in healthy tissues, nerves and some of their products regulate various mechanisms deeply involved in the carcinogenesis process, such as: control stem cell proliferation [26,27], angiogenesis [28], cell migration [29], cell translocation [30], cell differentiation [31], recruitment and activation of immune cells [32], and energy metabolism [33]. All of them are necessary mechanisms in proliferative physiological processes such as embryogenesis, healing or epimorphic regeneration. In addition, these normal physiological proliferative processes, are nerve-dependent. When nerves are absent, they alter, delay, or these processes simply do not occur [27,34]. 

In the case of angiogenesis, it is known that nerves and blood vessels are closely related in structure and organization, both during embryonic development as in response to damage, and both depend on bidirectional signals for self-regulation [35]. In various tumors, it has been possible to identify areas with intratumoral nerve fibers, located by immunohistochemistry [36,37,38,39]. These fibers are frequently found on the periphery of tumors, some in the stroma and others in a perivascular manner, however, some blood vessels are devoid of innervation, in our opinion this could cause failure in cellular communication between both elements. Consequently, the angiogenic process would be deregulated and this could partially explain the irregular formation of blood vessels and their heterogeneity within a tumor [40]. 

## 4. Evidence of the Involvement of the Nervous System in Cancer

Research on the neurobiology of cancer is so far scant. However, today there is greater interest and acceptance due to the evidence that is now available. For instance, epidemiological studies report a correlation between neuroactive therapies (benzodiazepines, lithium, or tricyclic antidepressants) with a reduced risk of cancer [41]. There is also an inverse correlation between neurodegenerative diseases and cancer. A good example is the case of patients with Parkinson’s disease or Alzheimer’s disease, where a low risk of cancer was identified, both before and after diagnosis [42]. 

Cognitive damage is a frequent event in cancer patients, and in some cases, diffuse brain damage has been identified through imaging studies. It is not clear whether this damage can occur as an adverse effect of anti-cancer therapies or neurodegeneration due to oxidative damage, inflammation and/or age effect associated with the oncologic disease [43]. Another interesting finding is the one that has been seen in patients with spinal cord damage and prostate denervation, who have a lower incidence of prostate cancer [44].

Other studies in patients with breast cancer have reported that women who use beta-blockers incidentally for some cardiovascular disease, before and/or during the year of breast cancer diagnosis, show a reduction in progression and mortality or remain free of the disease for a longer period, compared to patients who did not undergo beta-blocker treatment [45,46,47]. It is relevant to highlight that the use of beta-blockers seems to be more efficient in triple-negative breast tumors, which represents an option to be studied as a therapeutic target for this subtype of breast tumor, which has a highly aggressive biological behavior and so far, and does not show fully efficient treatment options [47]. 

The parallel benefit of the use of beta-blockers has been observed in complementary treatments in patients with other types of cancer, such as melanoma [48], pancreatic [49] and lung cancer [50]. In addition, it has been experimentally observed that treatments with beta-blockers in conjunction with other therapies, such as chemotherapeutics [51], immunotherapies with αPD-1 [48], or metformin [52], experimentally prevent the tumor growth and its progression. On the contrary, the action of catecholamines, when using adrenergic agonists such as isoproterenol, increases the occurrence of metastases to distant tissues compared to control tissues [9]. 

The development of metastases, via the hematogenous and/or lymphatic pathways, has long been considered the most relevant mechanism in malignancy and the leading cause of mortality in cancer patients. More recently, the neural pathway of metastasis was described and called perineural invasion (PI). Perineural invasion is considered a strong factor indicative of malignancy and has been associated with the worst prognosis [53]. 

Infiltration of neural tissues has been observed in various types of tumors, such as pancreatic [54,55], squamous cell carcinomas [56], and colorectal carcinoma [57,58,59], among others. Although the mechanisms by which this phenomenon occurs are not entirely clear, in vitro studies have shown that nervous and tumor tissue can have mutual tropism and exert chemoattraction, since the nervous tissue releases signaling molecules that guide tumor cells towards them [60,61].

## 5. Tumor Innervation

The process of forming new nerve fibers is called axonogenesis. The mechanisms by which tumors become innervated are still to be clarified, but for this new formation to occur, the confluence of various stimuli that include growth factors, neurotrophins, cytocines, and axonal guide molecules is necessary, resulting in the differentiation of neural precursors and their development or elongation. Various factors related to the development and activation of the nervous system have been found to be highly expressed in tumors, such as pro-NGF, NGF, BDNF, NT, VEGF, FGF, TGFs, semaphorins, and netrins [41,62,63,64]. These neurotrophic factors could be the inducers of the new formation of nerve fibers within tumor tissues. It is known that they can be produced by the same tumor cells and/or inflammatory cells, exert autocrine or paracrine stimulation, and activate signaling pathways through their action on TrkA and p75NTR receptors. The activation of these receptors leads to the stimulation of cell proliferation and cell survival [65].

The possible ability of tumors to stimulate their own innervation as occurs with angiogenesis and lymphangiogenesis, has been defined as neoneurogenesis [64,66]. Although the presence of nerve fibers has been described in various types of tumors, neoformation has only been described in prostate cancer [67]. Hyperinervation occurs in pancreatic tumors that could be partially dependent on β2ADR signaling [49]. Interestingly, a recent study has explained the presence of prostate tumor cells that presented expression patterns similar to those found in the subventricular zone (SVZ) and when isolated they were able to proliferate and differentiate into neurons, thus suggesting that the adrenergic neoformations observed in tumors may originate in situ from progenitor cells that could reach the tumor from neurogenic areas of the brain [68]. However, other substances could influence the modification in the number of nerve fibers present in tumors, for example, we can find physiological nerve plasticity in reproductive organs due to variations in circulating hormonal concentrations [69], therefore, it could be possible that some tumors stimulated by hormones such as breast tumors, may suffer nerve plasticity due to the presence of hormones in their microenvironment.

## 6. Nerve Diversity in Varied Tumor Types

One question about tumor innervation is, if it is similar to the innervation in the primary tissue where it originated, or if the tumor innervation is totally independent and established according to the needs of the new neoplastic tissue. 

The prostate and pancreas are highly innervated organs, and their function responds to a large extent to nervous stimuli, likewise, the colon presents high nerve density and it is in primary tumors of these organs that the innervation of neoplastic tissues has been studied the most, seeking their presence and effect [49,67]. 

Perivascular denervation of sensory fibers (NPY, VIP, SP) and adrenergic fibers of blood vessels present in the stroma and the submucosa adjacent to the tumor have been observed in colon cancer studies. The greatest loss occurred in TH^+^ and NPY^+^ fibers from tumors that showed a higher degree of malignancy, which raises potential temporary modifications [39]. It is suggested that nerve degeneration is the result of the release of tumor factors and that it could lead to bowel dysfunction [39,70]. In a study with a large number of samples (*n* = 236) using tissue array slides immunostained for protein gene product 9.5 (PGP9.5) to identify nerve tissue, Albo et al. [71] observed PGP9.5+ nerves in the stroma of colon adenocarcinomas in 67% of the cases, being 11% considered as having high nerve density (>20 nerves per hpf). Moreover, rectal tumors showed a high degree of innervation (73%), of which 20% showed high nerve density. Patients with high nerve density had a lower 5-year survival rate in comparison with those who did not show the presence of nerves (40% vs. 86%) and high recurrence and median survival of 2.8 years vs. 9.7 years. For this reason, nerve density was considered a powerful independent prognostic factor, of greater relevance than observing positive lymph nodes [71].

In the case of breast tumors, at first, the presence of nerve fibers was reported only at the junction between healthy tissue and neoplastic tissue, without evidence of perivascular innervation, and associated with well-differentiated tumors [36]. Twenty years later, in another study, nerve fibers were observed in the tumor stroma and around blood vessels; high nerve density was then associated with tumors with a higher degree of malignancy [37]. Although both studies present contradictory results, they cannot be considered determining or misleading, since reports and information on the matter are still scarce.

In a recent study, the association between perineural invasion (PNI) and locoregional recurrence (LRR) accounting for age, tumor size, nodal involvement, estrogen receptor, progesterone receptor, HER2 status, histologic tumor grade, presence of lymphovascular invasion (LVI), and receipt of chemotherapy and/or radiation among a broadly representative cohort of breast cancer patients was evaluated [72]. Their analyses revealed that PNI represents a significant risk factor associated with LRR following the definitive treatment of invasive breast cancer, and must be considered among other clinicopathologic features, such as young age, lymphovascular invasion, and high-grade tumor [72].

The presence of peripheral innervation has been found in other types of tumors, as well as the presence of neurotrophic factors that could benefit them, such is the case of ameloblastomas [73], papillary thyroid carcinoma [74], and among others, some benign tumors such as cutaneous neurofibromas, where a greater number of nerve fibers was found in the evaluated neoplastic tissues, compared to the control tissue [75]. For the most part, the abundance of nerves in newly formed tissues is associated with malignancy and the nerve phenotypes mainly reported correspond to adrenergic innervation, although it is important to note that there is nerve variation in the different histological classifications of primary tumors of the same cell line.

## 7. Neurotumoral Communication

The interaction of neural elements with tumor cells is defined as neuro-neoplastic synapses. The nerves in the tumor parenchyma, through the release of neurotransmitters, can interact with membrane receptors on tumor cells and other types of cells present in the stroma, for example, endothelial cells, leukocytes, and fibroblasts [76]. Neurotumoral synapses can also occur in CNS tumors, such as gliomas. Gliomas form neurotumoral synapses through a presynaptic neuron and a postsynaptic tumor cell, and these synapses can be as functional as synapses formed by neurons. Additionally, the presence of AMPA receptors (AMPARs) in glioma cells of the postsynaptic region was also reported [77]. AMPARs are ionotropic glutamate receptors that are expressed throughout the CNS and regulate most of the fast-excitatory transmission. The changes that occur at AMPARs synapses lead to different forms of synaptic plasticity that trigger some of the important mechanisms involved in learning and memory formation [78].

Neurotumoral communication could occur through two pathways, the humoral and the neural, with an important function of the peripheral autonomic and sensory nerves [79]. Although it is mentioned that the hematogenous pathway does not turn out to be the main provider for the delivery of neural stimuli towards a tumor [80], the transport of stem cells and neural progenitors from the subventricular area of the brain, capable of migrating and colonizing selectively a tumor and metastasis sites, occurs through blood vessels. These progenitor cells can then differentiate into adrenergic neurons [65], and therefore, the innervation of a tumor can occur from within itself. On the other hand, some neurotransmitters may not originate from nerve cells and be synthesized in situ from other stromal cells, such as lymphocytes, macrophages, and neutrophils, since these have the necessary machinery for synthesis, release, and inactivation of catecholamines, in fact, the phagocytic system is considered a diffuse adrenergic organ [81].

**Figure 1:**
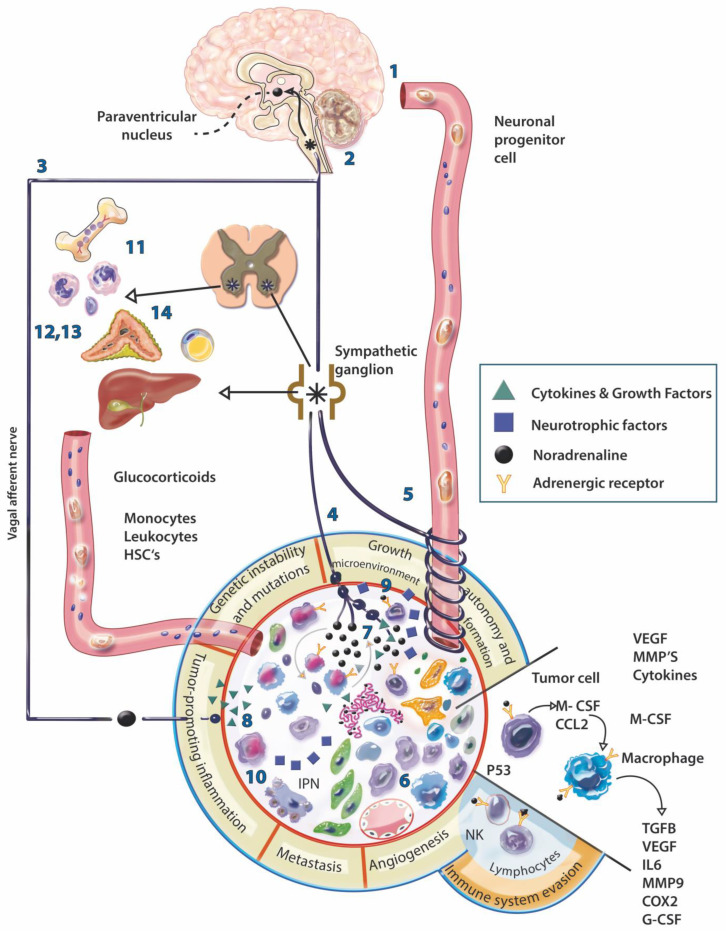
Physiological and neural modulation of the tumor. The communication of the nervous system (NS) with the tumor can be direct, through the hematogenous (1) or neural route (2), or indirect through the stimulation of other organs (3); this communication could be bidirectional. Nerve fibers can be present in the periphery of the tumor, perivascularly, even in the stroma (4), although they are generally scarce, the fibers present dilations that could amplify the signal several nanometers away, activating membrane receptors of various cell types present in the stroma. The direct influence of the NS on the tumor involves: the migration of progenitor cells through the blood from the paraventricular nucleus (PVN) to the tumor (5), to subsequently differentiate into nerve cells; increased infiltration by macrophages and MDSC cells in the tumor (6); the release of neural products in the tumor (7); the increased expression of COX-2, CSF-1, TGFb, MMP9, VEGF, VCAM1 and the decrease in IFNb by adrenergic activation of macrophages within the tumor (8); the release of neurotrophic factors such as NGF, BDNF and FGF by tumor cells that can induce neurite outgrowth (9) and perineural invasion (10). Indirectly, adrenergic stimulation is capable of: mobilizing bone marrow cells for subsequent recruitment into the tumor stroma (11); decreasing the mitotic activity and differentiation of lymphocytes and the proliferation and cytotoxicity of NK cells (12); promoting the trafficking of phagocytic cells (13); mobilization of energy from adipose tissue and liver (14) for later use by tumor cells. In addition, according to the physiology of a healthy individual, the increase in circulating glucocorticoids induced by stress causes a decrease in insulin levels, which results in an increase in circulating glucose generating a protumoral effect. * Sympathetic neurons.

The release of neurotransmitters in tumors can occur as a consequence of a multidirectional communication between the NS, the immune system, and tumor cells [80]. For example, cytokines released by inflammatory and/or tumor cells could cross the blood-brain barrier or transmit signals to the brain through afferent nerves, inducing structural and functional changes in the brain [82], which could trigger a homeostatic imbalance and influence the development of a neoplastic process. In this way, the central nervous system could not only promote the progression of the disease, but also monitor the processes of carcinogenesis initiation. Peripheral innervation would also intervene in an important way in this communication, this being the means for the stimuli to reach their destination, in addition, peripheral innervation controls various cellular phenomena similar to those observed in tumors, so it would not be strange that innervation assumed a central role in the pro and/or anti-carcinogenic mechanisms in the different stages of the disease (Figure 1).

## 8. Neuromodulation of Immunity

Both the Nervous System and the Immune System share soluble mediators and receptors in common, for example, leukocytes have receptors for neurotransmitters and neurons have Toll-like receptors (TLR), this is how communication occurs between systems and the brain can detect when an inflammatory process is occurring. In general, we know that the SNS promotes the humoral immune response and inhibits the cellular immune response by decreasing the cytotoxic function of T lymphocytes and NK cells [83]. The general conception of the functioning of the SNS and the PNS is that they have antagonistic effects, but in fact, they work together. During physiological processes, both exert coordinated and dependent functions on one another for the maintenance of homeostasis [84].

In the inflammatory process, sensory nerves pick up inflammatory signals during the innate immune response and release neurotransmitters and neuropeptides such as SP, and CGRP in response to damage. The release of pro-inflammatory mediators is set off and the inflammatory response is triggered, which includes vasodilation, vascular permeability, and pain. During this process, signals produced can be felt by the CNS, through the uptake of cytokines, prostaglandins, and damage molecules that can cross the blood-brain barrier, or by receiving an afferent signal captured by the vagus nerve [84]. 

Subsequently, through an efferent signal, the peripheral ganglia release acetylcholine to stimulate peripheral nerves that release noradrenaline, this adrenergic activity in T lymphocytes causes them to release acetylcholine and by cholinergic activation, the production of TNFα, IL-1β and IL-12 is modulated in macrophages [83]. T lymphocytes will now produce INFγ, achieving an anti-inflammatory effect [84] for the modulation of the inflammatory process, bringing the tissue to a homeostatic balance. Some populations of NK cells also have the ability to synthesize acetylcholine, either by neural stimulation or by an inflammatory process, overexpressing genes related to cytokines and chemokines involved in the regulation of the immune response and chemotaxis during the inflammatory process, and underexpress genes related to cytotoxicity. The local effect of positive NK cholinecetyltransferase (ChAT+) cells is the decrease in the migration of infiltrating monocytes/macrophages and the presence of pro-inflammatory cytokines [83]. Another anti-inflammatory mechanism, which is regulated by the vagus nerve, is the release of dopamine in the adrenal glands; this effect has been observed in experimental sepsis models stimulated with electroacupuncture, which manages to modulate the production of catecholamines [85].

Adrenergic stimuli, on the other hand, influence the migration of immune cells locally in the initial phase of inflammation, regulated by α-receptors. In the late stages, adrenergic signals have an anti-inflammatory impact and are regulated by β-receptors. Noradrenaline has anti-inflammatory effects at high concentrations and, conversely, at low concentrations, it exerts a pro-inflammatory effect [85].

Both macrophages and precursor monocytes express β-adrenergic receptors. The stimulation of these receptors in macrophages produces the synthesis of different cytokines such as IL-6, IL-10, and TNFα. It also modulates the organization and dynamics of actin and the deformation of macrophages to induce phagocytosis and migration [86] (Figure 1). 

## 9. Intrinsic Properties of Malignant Tumors and Their Mechanisms Related to the Nervous System

**Figure 2:**
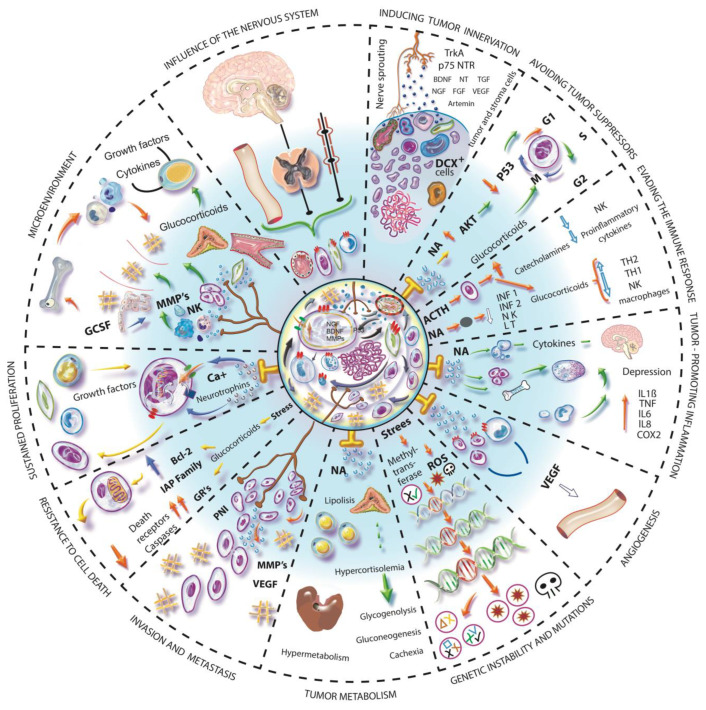
Influence of the nervous system in hallmarks of cancer. The nervous system by its pleiotropic effect can influence different mechanisms involved in the hallmarks of cancer. Communication occurs via hematogenous or neural pathways and stimulates different types of cells inside and outside the tumor. INDUCING TUMOR INNERVATION. Tumor and stromal cells release various neurotrophic factors that induce peripheral nerves to innervate the tumor. Furthermore, DCX^+^ cells can differentiate and generate tumor innervations. AVOIDING TUMOR SUPPRESSORS. The adrenergic stimulus causes an increase in cAMP and activation of AKT, which results in the ubiquitination of p53 and its subsequent degradation, which will cause the accumulation of DNA damage and the maintenance of cell division. EVADING THE IMMUNE RESPONSE. Both the HPA axis and the SNA regulate the response to stress, the consequent release of glucocorticoids and catecholamines have an immunosuppressive effect, suppress the production of proinflammatory cytokines and improve the production of anti-inflammatory cytokines. TUMOR-PROMOTING INFLAMMATION. The mobilization of monocytes towards the blood circulation depends on the local adrenergic activity of the bone marrow, in addition, CSF-1 increases its expression by adrenergic activity in macrophages and favors its recruitment in the tumor. Proinflammatory cytokines can activate the HPA axis. Cytokines released by chronic stress promote depression-like behavior by disrupting the synthesis and signal transduction of neurotransmitters. ANGIOGENESIS. Adrenergic stimulation and chronic stress favor the expression of proangiogenic factors and some sympathetic nerve fibers can release NPY. GENETIC INSTABILITY AND MUTATION. Stress-related hormones can cause DNA damage and promote cell transformation through the production of ROS, expression of DNA damage genes, and decreased DNA repair. The decrease in methyltransferase due to stress favors gene expression and facilitates cell transformation and genotypic diversity. TUMOR METABOLISM. Adrenergic stimulation produces energy mobilization from the liver, increases glycogenolysis and gluconeogenesis in hepatocytes, also inhibits insulin secretion from the pancreas, stimulates glucagon, and also increases lipolysis. These events favor the availability of energy for the tumor. INVASION AND METASTASIS. The modification of the microenvironment and the extracellular matrix, the formation of blood vessels and the presence of M2 macrophages, are important events in the development of metastases that can be induced by adrenergic activation. A perineural invasion is a form of metastasis in which communication occurs between the nerve fiber and tumor cells. RESISTANCE TO CELL DEATH. Glucocorticoids have an antiapoptotic effect and give survival signals to epithelial cells since they favor the expression of BCL-2 and IAP family proteins and prevent cell death. SUSTAINED PROLIFERATION. The neurotrophins that are released by tumor cells stimulate the nerve fibers that correspond to the release of neurotransmitters to activate the receptors of the cells present in the tumor, where the adrenergic stimulus can activate calcium channels and the mobilization of calcium activates IGF receptors that promote proliferation and tumorigenesis. In addition, the expression of neurotrophins by binding to their receptor induces proliferation and survival, an example of this is the binding of BDNF/TrkB. MICROENVIRONMENT. The microenvironment of a tumor is dynamic, resulting from extensive communication between tumor and stromal cells, inducing each other. The microenvironment is as important as the proliferation of tumor cells, it could even be more powerful due to the transformation capacity it has on epithelial cells. The nervous system may participate in the formation of the microenvironment of a tumor in a pleiotropic manner.

In 2000, Hanahan and Weinberg published the first hallmarks of cancer. In 2011, the same authors added another four elements to the model, which already considered the interactions of cancer cells with the stroma and recognized the latter as an active participant in the process of tumorigenesis. Today, 14 essential characteristics have been described that add up to the formation and progression of cancer [87,88,89]. The nervous system, directly or indirectly, can influence some of these necessary elements and other factors that have been found relevant in the development of cancer, for example, the modulation of stem cells, the stimulation of ion channels or the alteration of the microbiota [90]. Below, we describe how the nervous system could influence the main mechanisms involved in the formation and malignancy of neoplastic tissues (Figure 2). 

### 9.1. Evasion of Tumor Suppressors, Genetic Instability and Mutation

Stress has been associated with the progression and malignancy of cancer. However, some studies suggest that it can also facilitate genetic instability and mutation being involved in the early stages of the carcinogenic process [91]. 

The 8-hydroxydeoxyguanosine (8-OH-dG) is a marker of oxidative DNA damage, capable of causing mutagenesis and carcinogenesis, which occurs as a result of exposure to carcinogens and other hazardous materials. By examining the formation of 8-OH-dG in peripheral blood leukocytes in workers of a manufacturing corporation, it was determined that 8-OH-dG levels were associated with the perception of psychological stress and workload, being considered a risk factor for cancer, particularly in women [92]. 

It is well known that the formation of reactive oxygen species (ROS) causes DNA damage. The prolonged serum elevation of glucocorticoid levels can negatively influence mitochondrial function leading to mitochondrial damage with a negative impact on cellular metabolism and consequent inflammatory reaction [92]. All these observations have been drastically reduced through the administration of diazepam prior to a stressor stimulus and antipsychotic drugs such as olanzapine [90], which reduced the perception of stress in the individuals. This fact supports the idea that stress could be involved in preliminary steps for the initiation of the carcinogenic process by the alteration of cellular metabolism, tissue stress, consequent inflammation, the formation of ROS, and the consequent transformation of the healthy microenvironment in a pro-tumoral microenvironment.

In other studies, the possible effects of stress on tumor initiation have been studied. During the exposure of 3T3 fibroblasts to cortisol and noradrenaline for periods longer than 10 min, it was possible to observe a modification in the expression of genes related to the signaling pathways of DNA damage; also, the repair capacity and the regulation of the cell cycle were negatively affected, the apoptosis of the damaged cells was avoided, which eventually led to the transformation of cells. The effect of exposure to noradrenaline caused greater damage compared to glucocorticoids, damage that managed to be blocked with the use of propranolol or a glucocorticoid receptor antagonist [91]. These mechanisms could certainly contribute to the formation of precancerous lesions.

Another effect of adrenergic signaling that could be significantly involved in the cancer initiation stage is decreased p53 function, which occurs through β-arrestin-1-mediated AKT activation and Mdm2 phosphorylation and activation, leading to the nuclear export, ubiquitination, and degradation of the p53 tumor suppressor. Both, adrenergic stimulation and hypothalamic-pituitary-adrenal HPA axis stimulation can synergistically boost Mdm2 function and decrease p53 levels [93]. Stress also causes the decrease in methyltransferase, which would also affect the repair capacity of DNA [94]. 

All this suggests that the NS could be involved in chromosomal instability and evasion of tumor suppressors during the initial stages of tumorigenesis and that stress could also participate in the initiation process. For this reason, the emotional state can be considered a risk factor for cancer development and progression [91,93].

### 9.2. Evasion of the Immune System

The activation of the HPA axis occurs via afferent or by stimulation of inflammatory mediators and counteracts the effects of inflammation by the effect of glucocorticoids released from the adrenal cortex [95]. It is possible that this immunosuppressive activation could participate in the cell transformation process, where it is necessary for cells to escape cytotoxic destruction for the progressive acquisition of malignant characteristics. Glucocorticoids inhibit the availability and function of lymphocytes, NK cells and macrophages; regulate gene transcription and gene expression of mediators, receptors, adhesion molecules, and cytokines. The cytokines from which their synthesis is mainly inhibited are the IL-1, IL-2, IL-3, IL-4, IL-5, IL-6, IL-8, TNFα, IFN-γ, GM-CSF. It is also reported that glucocorticoids can increase the transcription of βARs in different tissues [96,97,98], which triggers vasoconstriction by adrenergic stimulation. 

In various studies, it has been observed that adrenergic stimulation has an immunosuppressive effect, for example, through T cell treatments with noradrenaline, epinephrine, or adrenergic agonists (such as isoproterenol or terbutaline). In vitro inhibition of the differentiation of naïve T lymphocytes to Th1 cells was also observed, by affecting early events involved in the initiation of proliferation, which may be explained in part by decreased IL-2 production by activated T cells. Likewise, the production of INF-γ in Th1 cells is decreased [99]. NK cells also express adrenergic receptors, the adrenergic activation of these cells inhibits their number and cytotoxicity. This fact could be verified in an experimental study by administering propranolol to mice stressed by sleep deprivation, increasing the number of NK cells and their cytotoxicity in vitro with melanoma cells [98]. Adrenergic signaling also promotes the activation and proliferation of myeloid-derived suppressor cells (MDSCs) and it has been proven that the use of propranolol can reverse these effects [100].

### 9.3. Tumor Associated Inflammation

The inflammatory cells that colonize the tumor microenvironment provide molecules that help maintain and support neoplastic cells. Molecules such as EGF, VEGF, FGF2, metalloproteinases, cytokines, and chymosins, make this particular characteristic an orchestrator of much of the rest of the essential features involved during carcinogenesis [101]. Some tumor cells through oncogenic activation are self-sufficient in the production of pro-inflammatory mediators such as cytokines, chymosins, prostaglandins and chemotactic factors, such as GM-CSF and G-CSF that favor the recruitment of inflammatory cells in the tumor and therefore the progression of solid tumors [102]. The immune system communicates in an important way with the nervous system, and this has the ability to influence pro-inflammatory and anti-inflammatory effects, which can undoubtedly favor the development of tumors.

Macrophages, endothelial cells, smooth muscle cells, and immune cells such as B and Th1 lymphocytes express β-adrenergic receptors. SNS affects clonal expansion, cytokine production, and target cell receptors expression; modifying the balance between the cellular and humoral immune response, increasing or inhibiting the response and mobilization of immunocytes. In macrophages, the response to adrenergic stimulation depends on the receptor that is activated. β-AR stimulation inhibits the activation of Ca^2+^-dependent K^+^ channels while α1-AR stimulation activates these Ca^2+^-dependent K^+^ channels. The mobilization of intracellular Ca^2+^ by adrenergic activation is important for the modulation of the phagocytic activity of macrophages and thus it can be explained that α-AR receptors regulate the improvement of phagocytic activity while β2-AR stimulation suppresses it [99,103]. In malignant tumors, myeloid cells and tumor-associated macrophages develop an immunosuppressed phenotype, which is regulated in part by TGFβ activity, and the stimulation of β-AR decreases the production of IL-6, IL-1β, IL-18, and increase in COX2 and CSF-1, the latter favoring the recruitment of macrophages in the tumor microenvironment [9]. 

The inflammatory process associated with a tumor participates not only in the recruitment and infiltration of differentiated inflammatory cells into the tumor parenchyma, but also in the mobilization of hematopoietic and progenitor stem cells (HSPCs) from the bone marrow for subsequent recruitment. The egress of HSPCs is regulated by G-CSF, which induces peripheral β-adrenergic signaling and allows the decrease in the chemokine CXCL12 in the bone, this decrease is what allows HSPCs mobilization [26,30]. Damage to hematopoietic regeneration and HSPC mobilization can occur due to sympathetic neuropathy in the bone marrow [95]. 

In some tumors, it has been possible to observe the joint participation of neural and immune elements. In induced hepatic tumors in rats, it was observed that Kupffer cells stimulated with LPS + noradrenaline significantly increase the expression of IL-6 and TGF-β compared to single induction with LPS. In addition, the blockade of the α1-ARs receptors caused the decrease in the expression of mRNA of CD90 [104], a protein associated with a higher incidence of metastases to distant organs [105], and a decrease in CD133, a marker of cancer progression [106]. Moreover, the activation of Kupffer cells and increase in cytokine levels was observed, which was related to the high density of nerve fibers present in the tumor [107]. So, it is possible that sympathetic innervation promotes carcinogenesis through the regulation of a pro-oncogenic inflammatory microenvironment. 

### 9.4. Angiogenesis

One of the most relevant mechanisms for cancer growth and the one responsible for the formation of one of the main pathways for the development of metastases is angiogenesis. Angiogenesis supports the maintenance and nutrition of tumor cells and helps the formation of the microenvironment by altering the extracellular matrix. Angiogenic factors are released mainly by tumor and endothelial cells; however, some sympathetic nerve fibers may release NPY, which is a potent angiogenic factor [108]. VEGF is the transcendental protein that promotes the proliferation of endothelial cells for the formation of new blood vessels. Chronic stress adrenergic activation induces VEGF expression in endothelial cells and in some types of tumor cells through the ADRβ2-PKA-CREB pathway [109,110]. CREB-induced HDAC2 has also been seen to epigenetically inhibit the antiangiogenic factor TSP1 in prostate cancer models [111]. Multiple cells in the tumor stroma can secrete proangiogenic factors, such is the case of macrophages that, by adrenergic stimulation, can express VEGF, MMP9 [9], and G-CSF, which is also capable of mobilizing endothelial progenitor cells from the bone marrow [102]. 

Dopamine is one of the various endogenous regulators of angiogenesis which can modulate microvascular hyperpermeability, angiogenesis, and probably tumor growth. Receptors for both dopamine and serotonin have been found in various tumor types such as colon, ovaries, breast, kidney, and pancreas. Dopamine, by occupying the D2 receptors, can inhibit angiogenesis, preventing the phosphorylation of VEGFR-2 from endothelial cells in tumor tissue and blocking the effect of VEGF, generating an antiangiogenic effect [112]. Furthermore, some in vitro studies demonstrated that Dopamine D1 receptor agonists suppress the proliferation of osteosarcoma OS732 cells, through the downregulation of the ERK1/2 and P13K-Akt pathways [113,114]. These data indicate that dopamine may have a protective effect. In malignant tumors, the existence of low concentrations of dopamine has been described. On the other hand, serotonin is associated with angiogenic development in malignant tumors [113]. 

Perivascular nerve fibers have been described in breast tumors [37], colon [71], pancreas, prostate, ovarian tumors, and melanoma, among others [115]. Vascular malformations have been identified in different tumor types and it was observed that they have smooth muscle around them [40]. From our perspective, it is possible that those vascular malformations could be innervated and provide vasomotor stimuli. 

### 9.5. Invasion and Metastasis

As mentioned above, the nerve fibers associated with a tumor can favor invasion and metastasis through the IPN, stimulate the mesenchymal epithelium transition, the synthesis of metalloproteinases, and confer positive survival signals to tumor cells, these mechanisms provide the morphology required for cell mobilization [110,111].

The immune system in its communication with the NS participates in this process and an example of this is the induction of the expression of pro-metastatic genes and genes characteristic of the M2 phenotype, such as TGFβ, in addition to the reduction in the expression of the IFN geneβ in macrophages located within the tumor microenvironment, in response to stress [9]. Additionally, β2ADR activation has a direct effect on tumor cells because the downstream signaling activates Src, regulated by PKA, and thus improves cell migration and invasion and growth of ovarian tumors [116]. Moreover, the β2ADR-cAMP-PKA pathway is involved in the activation of FAK, a focal adhesion kinase, implicated in cellular motility through the rearrangement of the cytoskeleton, increasing the phosphorylation of paxillin to improve the reassembly of F-actin and the remodeling of the extracellular matrix through the release of MMPs. Increased FAK has been observed in patients with metastatic prostate cancer and high levels of depression [117]. Similarly, there is evidence of reduction in metastases in experimental studies and in patients with various types of cancer, who have received treatment with selective and non-selective adrenergic antagonists, in a single treatment or combined with adjuvants, where, in addition, it has been found that the main regulator of these processes is β2ADR [52,110,118,119], however, it has also been reported that β2ADR agonists may have the same effect [120]. 

The NS can also be involved in conditions associated with metastasis which occurs during the neoplastic process. Such is the case of the common metastatic lesions to the bone that occur in breast cancer, where the invasion of tumor cells favors bone remodeling, causes osteolytic lesions, and induces outbreaks of sensory nerve fibers in the periosteum, in addition to histological disorganization and consequently bone pain [121]. Similar results were obtained in prostate cancer studies, where, in addition to sensory fibers, an increase in the density and disorganization of adrenergic fibers in bone tissue colonized by tumor cells, was observed [122]. In both studies, treatment with the anti-NGF antibody (mAb911) was administered, which was able to reduce nerve outbreaks and the consequent pain. One of the explanations for why breast cancer cells cause frequent metastases to bone is because sympathetic nerves abundantly innervate bone tissue and release noradrenaline to regulate remodeling and homeostasis. Osteoclasts express βARs and their stimulation causes the expression of RANKL, an osteoclastogenic cytokine with promigratory properties to cancer cells [123]. It should be considered that the lymphoid organs are also mainly innervated by the SNS, and it is where a large part of the invasion by tumor cells occurs, so it would be interesting to know if there is a predilection to areas where prometastatic colonization can be directed by neural influence.

The participation of the NS in cancer metastasis is not contemplated in the mechanisms accepted to date. However, the influence of the NS can occur through several regulatory mechanisms that could explain the frequent metastases to sites with no apparent explanation for their occurrence by the hematogenous route, such is the case of frequent metastases to bone (a tissue highly innervated) in different tumor types [124]. In our opinion, there is a need to consider the inclusion of NS as a regulator of metastasis; however, more research is needed.

## 10. Conclusions and Perspectives

For many years, cancer has been one of the most studied diseases worldwide; however, to date there is no cure for this clinical condition, so it is possible that some pieces of the puzzle are still missing in the study model. It is necessary to make a change in the approach from which it has been seen for so many years and to be able to take a turn in the understanding of this disease. 

As a result of several investigations, the need to expand and generate new knowledge in the cancer field has been widely recognized, but also in other areas that have been discarded for a long time and that could give interesting answers to integrate theories not very well accepted in their beginnings. One example of this is the Tissue Organization Field Theory (TOFT), where it is proposed that carcinogenesis is a relationship problem where reciprocal interactions occur between various cell types of the morpho-genetic field, and it is tissue disorganization and not autonomous uncontrollable cell proliferation that is the causal defect of cancer [125]. It is a reality that cancer is more than a proliferative disease, we have cellular and tissue diversity, a stroma, a microenvironment, diversity of substances, factors and molecules, all functioning at the same time for the benefit of the new tissue. It is also a fact that the nervous system has a pleiotropic effect and that is why it may be able to modulate multiple processes that occur during cancer, which is why further research is required to determine its degree of participation in the different stages and tumor strains.

Because the neurobiology of cancer has so far been little explored, but with solid evidence of its participation in the processes of carcinogenesis, the insertion of the nervous system in the study model is considered of high relevance. The plasticity and nerve remodeling of solid tumors, through various events such as axonogenesis, hyperinnervation or denervation at specific times of their development, activate or suppress the mechanism according to the requirements of the neoplasm, either for its formation or maintenance, similar to how it occurs in organogenesis. It is to be expected that the variation in their formation depends on the stage, grade and organ in which the tumors are located; there may be other factors that determine the timing, type, and density of nerve formation, such as metabolism, or the internal and external environment to which it is exposed. Finally, this information might answer what its biological function is in the process of carcinogenesis and how it could be used for the benefit of patients.

The nervous system represents a promising option for the understanding and, therefore, future treatment of cancer due to the pleiotropic effect it presents since it could attack different mechanisms at the same time. First, it is necessary to know what type of tumors are innervated and which are not, the phenotype of innervation present or absent, the anatomical location, its function or effect to which they are associated, and finally, understand the integration of how the association with tumor cells, their microenvironment and the organism occurs. It is relevant to visualize the possibility of generating future therapeutic targets, prognostic and/or early biomarkers of disease, and understand the possible etiopathogenesis of cancer contemplating all its parts.

With all the data collected in the multiple investigations carried out around the world which have allowed a deepening of the knowledge and understanding of its biology, it can be assumed with certainty that the nervous system has relevant participation in different stages of carcinogenesis by providing survival signals to tumor cells, promoting a favorable microenvironment for cell proliferation, and facilitating the migration of tumor cells, although the mechanisms are not yet fully defined. 

Cancer is a multisystem, multistage, multimechanism disease, so the deregulation of different biological-systemic processes that can impact the carcinogenic process must be considered. For the study of the neurobiology of cancer it is important not to forget the complexity of organisms and reject reductionism, contemplating that living beings are complex, multicellular organisms, with multiple internal and external agents influencing them, although there are genes or proteins that alone can generate disease and are importantly involved in the development of various pathologies, including cancer; the extensive complexity of the neoplastic process does not allow any of its parts to be excluded for study, understanding and possible prevention and/or cure. The knowledge we have about the interactions between the nervous system and tumors is still limited, interest and research in this branch of cancer biology are increasing and although there are currently no definitive conclusions, a promising future is envisioned.
